# Visual Tracking in Development and Aging

**DOI:** 10.3389/fneur.2017.00640

**Published:** 2017-11-30

**Authors:** Jun Maruta, Lisa A. Spielman, Umesh Rajashekar, Jamshid Ghajar

**Affiliations:** ^1^Brain Trauma Foundation, New York, NY, United States; ^2^Department of Neurosurgery, Stanford University, Stanford, CA, United States; ^3^Department of Rehabilitation Medicine, Icahn School of Medicine at Mount Sinai, New York, NY, United States

**Keywords:** attention, eye movement, ocular pursuit, pediatric, smooth pursuit, saccade

## Abstract

A moving target is visually tracked with a combination of smooth pursuit and saccades. Human visual tracking eye movement develops through early childhood and adolescence, and declines in senescence. However, the knowledge regarding performance changes over the life course is based on data from distinct age groups in isolation using different procedures, and thus is fragmented. We sought to describe the age-dependence of visual tracking performance across a wide age range and compare it to that of simple visuo-manual reaction time. We studied a cross-sectional sample of 143 subjects aged 7–82 years old (37% male). Eye movements were recorded using video-oculography, while subjects viewed a computer screen and tracked a small target moving along a circular trajectory at a constant speed. For simple reaction time (SRT) measures, series of key presses that subjects made in reaction to cue presentation on a computer monitor were recorded using a standard software. The positional precision and smooth pursuit velocity gain of visual tracking followed a U-shaped trend over age, with best performances achieved between the ages of 20 and 50 years old. A U-shaped trend was also found for mean reaction time in agreement with the existing literature. Inter-individual variability was evident at any age in both visual tracking and reaction time metrics. Despite the similarity in the overall developmental and aging trend, correlations were not found between visual tracking and reaction time performances after subtracting the effects of age. Furthermore, while a statistically significant difference between the sexes was found for mean SRT in the sample, a similar difference was not found for any of the visual tracking metrics. Therefore, the cognitive constructs and their neural substrates supporting visual tracking and reaction time performances appear largely independent. In summary, age is an important covariate for visual tracking performance, especially for a pediatric population. Since visual tracking performance metrics may provide signatures of abnormal neurological or cognitive states independent of reaction time-based metrics, further understanding of age-dependent variations in normal visual tracking behavior is necessary.

## Introduction

A moving target is visually tracked with a combination of smooth pursuit and saccades. Human visual tracking eye movement is an attention-dependent feat (1, 2) that takes years to develop, and its functional maturity is not achieved at least until mid-adolescence (3, 4). After maturity, tracking performance declines with senescence with observable changes taking place in subjects aged 50 or older (5–7). However, the knowledge regarding performance changes over the life course is based on data from distinct age groups in isolation using different procedures (8–10), and thus is fragmented. Therefore, we sought to describe the age-dependence of visual tracking performance using a standardized procedure (11) and a sample with a wide age range.

The visual and motor neural processing delay poses a critical challenge in visual interception of a moving target, during which the target is repositioned from where it was detected. It would thus seem inevitable that the gaze be always misdirected from the target as long as the target keeps moving. However, this outcome can be sidestepped when the target movement is predictable (1, 12). As such, the use of a circular target trajectory has advantages (13, 14). First, the target motion can be described with only two constants, a constant speed and radius, contributing to its predictability. Second, this two-dimensional periodic movement can continue indefinitely within the orbital range of the eye. These properties make the stimulus particularly suited for studying the processes required to maintain predictive visual tracking (15–20). In addition, circular visual tracking is less vulnerable to movement of upper eye lids, which contributes to recording artifacts, than one-dimensional vertical tracking while affording eye movement data in both horizontal and vertical dimensions (13).

There is a ubiquitous pattern of rising and falling of cognitive performance over the lifespan (21). In particular, simple visuo-manual reaction time [simple reaction time (SRT)], which is measured as the elapsed time to a key press after a visual cue presentation, shortens with age through childhood and gradually lengthens during adulthood (22–24). SRT performance also depends on attention (25, 26). Compared to visual tracking, age-dependent changes in SRT are better established with large sample sizes in the order of thousands. Thus, we additionally sought to replicate the finding in SRT to confirm procedural validity and to identify in the same sample cohort similarities and differences in the patterns of age dependence between visual tracking and SRT performances.

## Materials and Methods

### Subject Enrollment

We studied a cross-sectional sample of 143 subjects aged 7–82 years old (37% male) as part of a larger research project on mild traumatic brain injury (concussion). Impaired attention is a key symptom of concussion, and the utility of both circular visual tracking and SRT metrics has been suggested for concussion screening as objectively quantifiable measures (26–29). All testing was conducted at the Citigroup Biomedical Imaging Center at Weill Cornell Medical College (WCMC) in New York, NY, USA. The protocol was reviewed and approved by the WCMC Institutional Review Board. Prior to data collection, written informed consent by adult subjects, or legal guardians of minor subjects with the minors’ assent, was obtained in accordance with the Declaration of Helsinki. Subjects were recruited *via* flyers posted at various facilities including colleges, office buildings, hospitals, and community centers in and around the New York City area. Potential subjects were screened for eligibility through interviews conducted over telephone. Adult eligibility was based on the individual’s responses to screening questions, and pediatric eligibility on a legal guardian’s. Participation required a minimum age of 7 years, a high school diploma or equivalent for those over the age of 18 years, and normal (or corrected to normal) vision. Individuals were excluded for a prior history of traumatic brain injury (including concussion with loss of consciousness), substance abuse, a known neurologic disorder, or a known psychiatric condition (including attention deficit disorder). Family history of psychiatric disorders was not obtained. Ages were recorded in terms of years and months.

Of 187 (39% male) subjects enrolled, one discontinued participation. The remaining 186 subjects were tested for visual tracking and SRT performance (described below). Collected eye movement data were screened with an automated algorithm, and those from 43 subjects were deemed invalid for greater than 10% of missing data, artifacts associated with inadequate quality of calibration, or poor head stabilization during recording as evidenced by a large change in visual fixation records. Age distributions were not different between valid and invalid eye movement data (*p* = 0.38, two-sample Kolmogorov–Smirnov test); therefore, dropping invalid data did not produce an age-related selection bias. SRT data for all 186 subjects were considered valid, resulting in a total of 143 subjects with valid visual tracking and SRT data.

### Visual Tracking Test

We measured subjects’ eye movements while they tracked a target that moved in a predictable manner. The details of the methods were described previously (11). Briefly, subjects performed a circular visual tracking task on a video-based eye tracker integrated with stimulus-presentation (EyeLink 1000, SR Research Ltd., Mississauga, ON, Canada). The stimulus was presented on a 120-Hz LCD monitor (SyncMaster 2233RZ, Samsung, Seoul, South Korea). The stimulus consisted of a target of 0.5° of visual angle that moved clockwise on a black background along a circular path with a radius of 10° at 0.4 Hz, at a constant speed of 25.1°/s. A 9-point fixation calibration procedure was followed by a validation procedure, which had the gaze returned to the points of fixation used in calibration. The semi-automated testing sequence that included text and recorded audio instructions, a 2-cycle practice run, calibration, validation, and two 6-cycle test runs lasted approximately 5 min. Binocular gaze positions were sampled at 500 Hz. The gaze and target records were digitally stored for offline analysis. The task was performed in a normally lit room while subjects sat with their head stabilized by a chin-head rest. The visual acuity of each subject was confirmed to be normal or corrected-to-normal prior to testing using a handheld vision chart.

To characterize the stability of the gaze on the target, we evaluated the variability of gaze position error along axes orthogonal (radial) and parallel (tangential) to target movement [SD of radial errors (SDRE), SD of tangential errors (SDTE)]. The smaller the SDRE or SDTE value, the more precise the tracking. To characterize the central tendency of gaze position relative to the target, we evaluated the mean radial error and the mean phase error. A negative radial error indicated the gaze drawing a smaller circular trajectory than the target. A negative phase error indicated the gaze trailing the target or phase lag. We also computed the horizontal and vertical smooth pursuit velocity gains (H and V gains), which were the ratios between smooth pursuit eye velocity and target velocity. Smooth pursuit velocity amplitudes were obtained by differentiating the horizontal and vertical eye position data and fitting the desaccaded traces with a sine function of the stimulus frequency using fast Fourier transformation. A smaller gain indicated less precise tracking. To focus on high quality records, monocular data from the eye with the smaller SDRE value were pooled for further analyses (11). The data associated with the first stimulus cycle were not analyzed so as to discount the initial transient response to the target movement.

### SRT Test

We collected simple visuo-manual reaction time data using the SRT component of the Automated Neuropsychological Assessment Metrics Version 4 [ANAM4 (30)]. Other components of the ANAM4 library were not deployed. The stimulus was a large asterisk symbol presented at the center of a blank computer screen. The subjects were instructed to press a response key as quickly as possible each time the stimulus was presented. There were 40 trials in a test and the results were automatically analyzed by the software. The software discarded a response made in less than 130 ms after the cue presentation in analysis. We chose the mean and SD outputs to characterize the subjects’ performance to be consistent with the previous literature on age-dependence of reaction time (22–24). We also computed the coefficient of variance (CV) to obtain a normalized intra-individual variability metric since the SD depends on the mean (24).

### Statistical Evaluation

Age-dependent improvement and decline in performance were described with a quadratic regression model. The dependent variable of the model was a natural logarithm transformation of age in years plus one, so that a deceleration of changes with age could be accounted for and the transformed values would always be positive. This approach produced excellent fits for the inverted U-shaped relationships between age and age-grouped mean brain weights [(31); adjusted *R*-squared values: 0.97 for male and 0.96 for female; root-mean-square error <50 g] and for the U-shaped relationship between age and age-grouped mean SRTs reported by Koga and Morant [(22); adjusted *R*-squared value: 0.83; root-mean-square error: 7 ms]. Pearson’s correlation coefficient was used to examine the relationships between visual tracking and SRT performances. A two-sample *t*-test was used to examine the effects of the sex of the subjects. The alpha level was set at 0.05.

## Results

**Figure 1:**
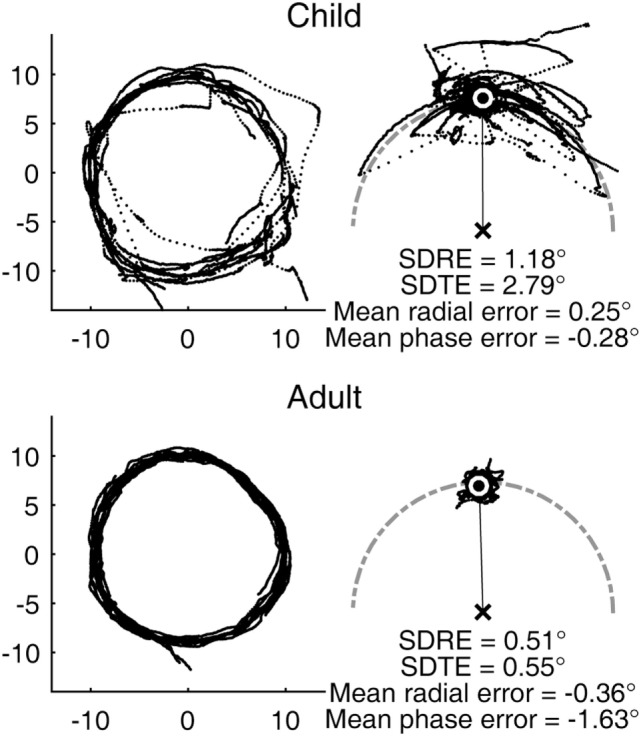
Representative circular visual tracking performances by child and adult. (Top) Performance of a 7-year-old boy. (Bottom) Performance of a 30-year-old woman. The left panels show the two-dimensional gaze trajectories of the respective subjects with each dot representing a sample taken at 500 Hz. The right panels show the same data with the gaze positions plotted in target-based reference frame, with the target fixed at the 12 O’Clock position. The center of the white circle indicates the average gaze position. The gray, dot-dashed curve indicates the circular path. The child subject had H and V gains of 0.78 and 0.58, respectively. The adult subject had H and V gains of 0.90 and 0.83, respectively.

The youngest subject in our sample was a male aged 7 years and 8 months. His visual tracking performance was characterized with frequent large saccades and highly variable gaze error relative to the target (Figure 1, top). His smooth pursuit gain was reduced compared to that of a typical adult. Still, his average gaze position fell very close to the target without any substantial lag. The performance of a typical adult subject was substantially more precise in comparison, although still not completely smooth, as expected from a biological system (Figure 1, bottom).

**Figure 2:**
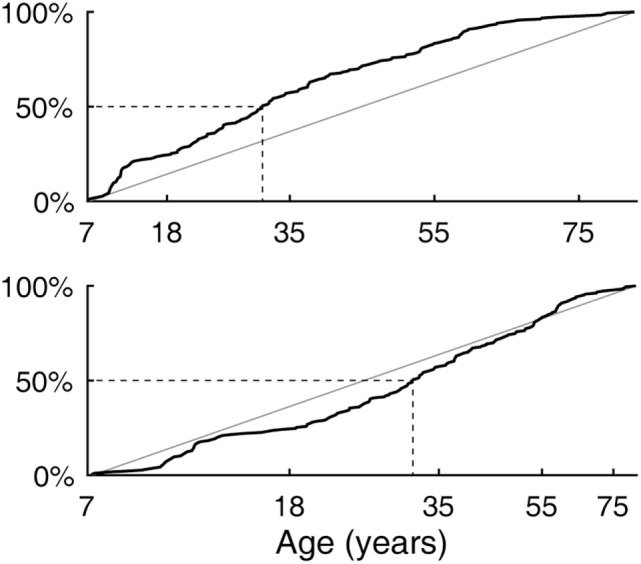
Subject age distribution. (Top) Empirical cumulative distribution as a linear function of age. (Bottom) Empirical cumulative distribution as a logarithm-based function of age with which quadratic regression fits were made.

The age difference between the youngest and the oldest subjects spanned 75 years and 6 months. The cumulative distribution of ages (Figure 2, top) rose slightly more steeply in the lower range, reaching the median at 31 years and 3 months of age. The output of the logarithm-based transformation of age was more uniformly distributed (Figure 2, bottom), supporting the validity of the subsequent quadratic regression fits. A strong bias toward any age band was not indicated.

**Figure 3:**
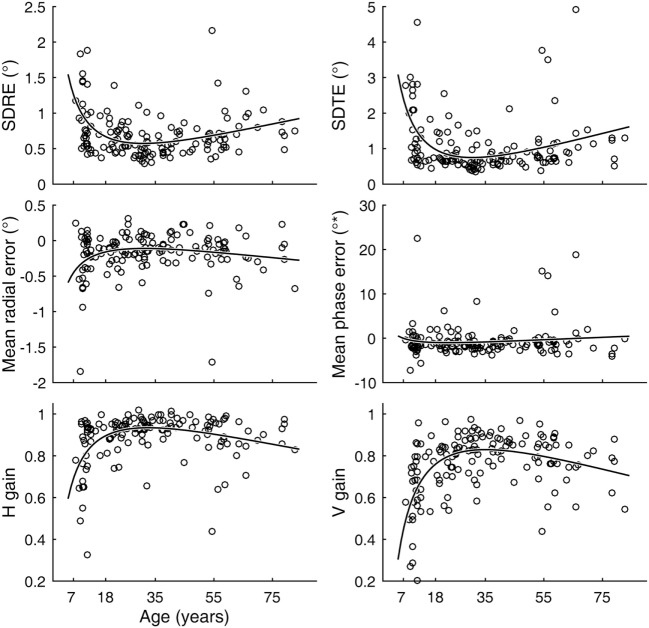
Visual tracking performance as a function of age. Circular markers indicate individual scores. Black curves indicate regression model fits. SD of radial errors (SDRE), SD of tangential errors (SDTE), and mean radial error are expressed in degrees of visual angle (°) while mean phase error is in degrees of phase angle (°*). H and V gains are dimensionless quantities. See Table 1 for model summary statistics.

Inter-individual variability in visual tracking performance was large across ages. Nevertheless, when visual tracking measures were plotted as a function of age, a U- or inverted U-shaped trend was evident for the SDRE, SDTE, and H and V gain metrics (Figure 3). We tested the trends with a quadratic regression model of the data as relating to a function of age (Figure 3, black curves). The model fits were statistically highly significantly different from a constant model for these metrics (Table 1). The troughs in the fitted curves of SDRE and SDTE occurred at ages 30.8 and 30.5 years, respectively. The peaks in the fitted curves for H and V gains occurred at ages 32.6 and 35.1 years, respectively. Additionally, a marginally significant difference from a constant model was found for mean radial error.

**Table 1:** Summary quadratic regression model statistics of visual tracking performance relative to a function (*X*) of age.

	B	SE	*t*	*p*-Value		B	SE	*t*	*p*-Value
**SD of radial errors**					**SD of tangential errors**				

*X*^2^	0.362	0.077	5.038	0.000	*X*^2^	0.879	0.176	4.997	0.000
*X*	−2.503	0.478	−5.238	0.000	*X*	−6.065	1.170	−5.182	0.000
Constant	4.902	0.776	6.314	0.000	Constant	11.215	1.902	5.897	0.000
*R*-squared value: 0.185					*R*-squared value: 0.180				
Adjusted *R*-squared value: 0.174					Adjusted *R*-squared value: 0.168				
*F*-statistics vs. constant model: 15.9, *p* = 0.000					*F*-statistics vs. constant model: 15.3, *p* = 0.000				

**Mean radial error**					**Mean phase error**				

*X*^2^	−0.176	0.071	−2.482	0.014	*X*^2^	0.861	0.914	0.942	0.348
*X*	1.222	0.472	2.590	0.011	*X*	−5.421	6.085	−0.891	0.375
Constant	−2.233	0.767	−2.912	0.004	Constant	7.527	9.887	0.761	0.448
*R*-squared value: 0.054					*R*-squared value: 0.008				
Adjusted *R*-squared value: 0.041					Adjusted *R*-squared value: −0.006				
*F*-statistics vs. constant model: 4.02, *p* = 0.020					*F*-statistics vs. constant model: 0.597, *p* = 0.552				

**H gain**					**V gain**				

*X*^2^	−0.119	0.025	−4.676	0.000	*X*^2^	−0.169	0.030	−5.686	0.000
*X*	0.833	0.169	4.937	0.000	*X*	1.211	0.198	6.126	0.000
Constant	−0.530	0.274	−1.931	0.056	Constant	−1.342	0.321	−4.177	0.000
*R*-squared value: 0.188					*R*-squared value: 0.304				
Adjusted *R*-squared value: 0.176					Adjusted *R*-squared value: 0.295				
*F*-statistics vs. constant model: 16.2, *p* = 0.000					*F*-statistics vs. constant model: 30.6, *p* = 0.000				

*X is the natural logarithm of age plus one. The coefficients of the terms in the quadratic fit to the transformed data are notated under B. The degrees of freedom for the *F*-statistics were (2, 140)*.

**Figure 4:**
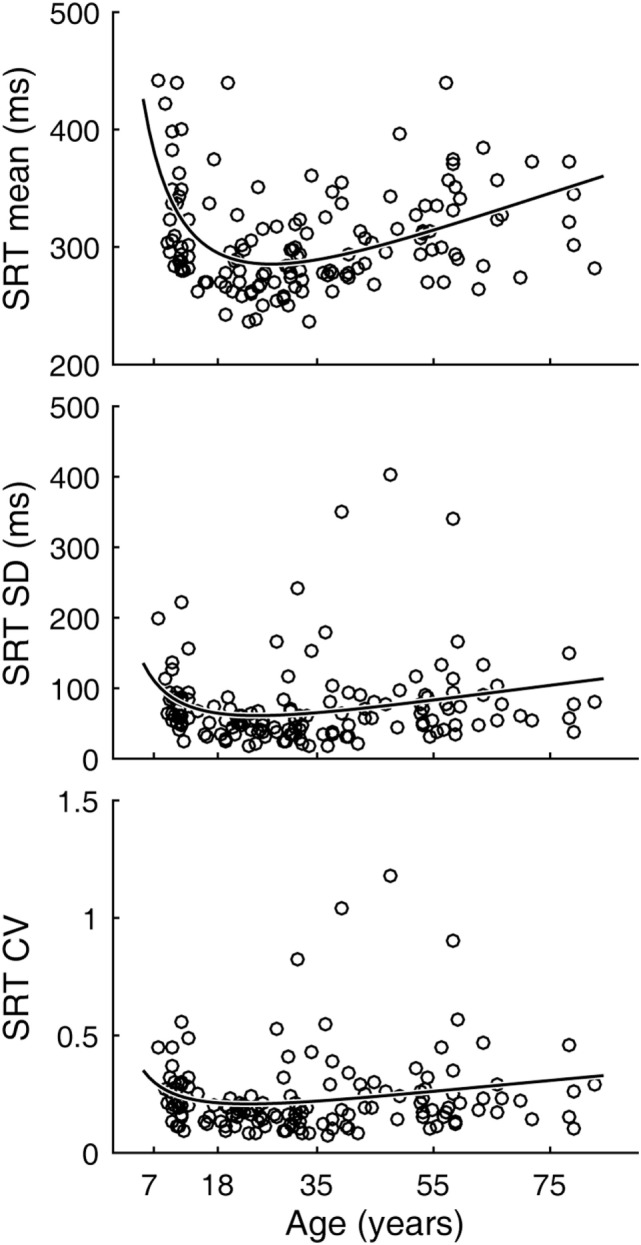
Simple reaction time (SRT) performance as a function of age. Circular markers indicate individual scores. Black curves indicate regression model fits. See Table 2 for model summary statistics.

Inter-individual variability in SRT performance was also large. However, consistent with previous knowledge (22–24) and similar to visual tracking performance, a U-shaped trend was contained within such inter-individual variability, most evidently for mean latencies (Figure 4). The quadratic regression model fit of the mean latency data (black curve) was statistically highly significantly different from a constant model (Table 2). The trough in the fitted curve occurred at 27.1 years of age. For SDs of latencies, the model fit was only marginally different from a constant model, and for CVs the model fit was not significantly different from a constant model.

**Table 2:** Summary quadratic regression model statistics of simple reaction time performance relative to a function (*X*) of age.

	B	SE	*t*	*p*
**Mean latency**				
*X*^2^	61.1	9.6	6.385	0.000
*X*	−407.5	63.7	−6.402	0.000
Constant	965.3	103.4	9.334	0.000
*R*-squared value: 0.226				
Adjusted *R*-squared value: 0.215				
*F*-statistics vs. constant model: 20.5, *p* = 0.000				

**SD of latency**				
*X*^2^	36.6	14.1	2.591	0.011
*X*	−237.6	94.0	−2.528	0.013
Constant	446.9	152.7	2.927	0.004
*R*-squared value: 0.048				
Adjusted *R*-squared value: 0.035				
*F*-statistics vs. constant model: 3.55, *p* = 0.031				

**Coefficient of variance**				
*X*^2^	0.076	0.041	1.856	0.066
*X*	−0.487	0.274	−1.778	0.078
Constant	0.984	0.445	2.213	0.029
*R*-squared value: 0.029				
Adjusted *R*-squared value: 0.015				
*F*-statistics vs. constant model: 2.07, *p* = 0.13				

*X is the natural logarithm of age plus one. The coefficients of the terms in the quadratic fit to the transformed data are notated under B. The degrees of freedom for the *F*-statistics were (2, 140)*.

Having established age-dependency in both visual tracking and SRT performances, we next looked for interdependence between the two. Specifically, we tested for within-individual correlations between visual tracking and SRT metrics after subtracting the estimated effects of age. No meaningful correlation was found (Table 3).

**Table 3:** Interdependence between visual tracking and simple reaction time metrics.

	SD of radial errors	SD of tangential errors	Mean radial error	Mean phase error	H gain	V gain
Mean latency	0.001	0.014	0.018	−0.048	−0.014	0.091
SD of latency	−0.105	−0.039	0.067	−0.052	0.040	0.017
Coefficient of variance	−0.116	−0.051	0.063	−0.051	0.046	0.007

*Pearson’s correlations between distances from age-based expectations (residuals) were calculated*.

**Figure 5:**
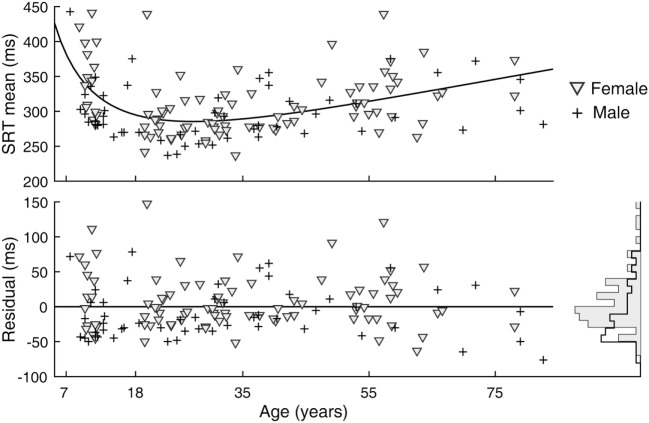
Sex difference in simple reaction time (SRT) performance. (Top) Duplicate of Figure 3, SRT mean, except with separate markers for female (gray triangle) and male (cross). (Bottom) Residuals of model fit for female and male subjects and their corresponding histograms.

**Figure 6:**
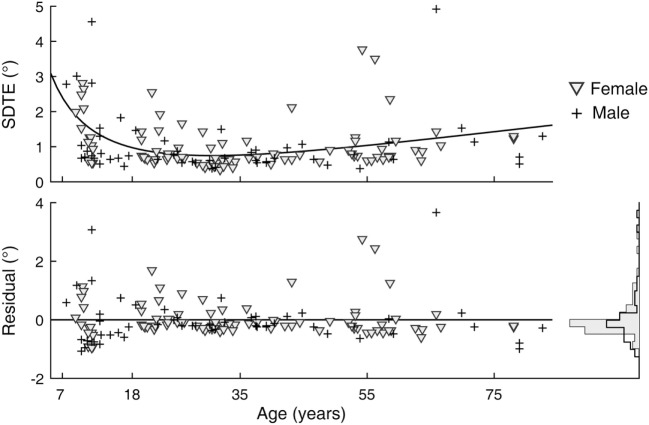
Sex difference in visual tracking performance. (Top) Duplicate of Figure 2, SD of tangential errors (SDTE), except with separate markers for female (gray triangle) and male (cross). (Bottom) Residuals of model fit for female and male subjects and their corresponding histograms.

A sex difference in SRT is a known effect, with males across the life span averaging faster reaction times than females (24). We observed this effect in our data as well (Figure 5). After subtracting the estimated effects of age from mean latencies, the residuals for female subjects were larger than those for male subjects [*t*_(141)_ = 2.64, *p* = 0.009]. A significant sex difference in the SD or CV metrics was not revealed by the same analysis. In contrast to SRT, none of the six visual tracking metrics yielded statistical significance; thus, there was no finding of sex differences in visual tracking performance. Shown in Figure 6 is a representation of the analysis conducted for the SDTE metric [*t*_(141)_ = 0.48, *p* = 0.63].

## Discussion

In a cross-sectional sample of normal individuals with an age range spanning from 7 to 82 years, we characterized age-dependent improvement and decline in performance on a standardized predictive visual tracking task. Large inter-individual variability in visual tracking performance was found across ages, but fastest developments occurred in young children. Changes in performance again accelerated in senescence, but this time as decline and at a slower rate than those in childhood. A notable exception to this pattern of improvement and decline was in the mean phase error metric, demonstrating an overall tracking timing accuracy across all ages presently studied. Given the visuomotor processing delay of some 100 ms (32, 33), a purely reactive mode of tracking on the present task should result in the gaze lagging approximately 14° behind the target. However, none of the subjects had the mean phase lag exceeding this value. Likewise, it has been reported that in normal adults, low fidelity of visual tracking performance is more strongly associated with the presence of anticipatory saccades than catch-up saccades (11, 34), illustrating a robust involvement of prediction in the tracking behavior. The predictive capacity in visual tracking develops rapidly within the first year of life (19), and our results indicate that this capacity is maintained well into old age.

In the same sample cohort, we were able to replicate the essential findings of large-scale studies that showed age-dependent improvement and decline in SRT performance as well as a sex difference (22–24). This benchmark supported the procedural validity of the present study and also allowed us to look for interdependence between visual tracking and SRT performances. Both visual tracking and SRT performances show within-individual stability over a time frame of weeks, bearing biometric characteristics (11, 34–36). Visual tracking and SRT performances are also both attention-dependent (1, 2, 25, 26), granted attention is multi-faceted (37, 38). Therefore, interdependence between performance characteristics between the two tasks should indicate shared neurological bases for individual variations. However, correlations were not found between visual tracking and SRT performances after subtracting the effects of age. Furthermore, while a statistically significant difference between the sexes was found for mean latencies in the SRT task, a similar difference was not found for any of the visual tracking metrics. Although it is possible that a subtle sex difference in visual tracking could be found under a different stimulus condition (34, 39) or with a larger sample size, taken together our results suggest that the cognitive constructs and their neural substrates supporting predictive visual tracking and SRT performances are largely independent. Such a separation may point to differences between predictive and reactive natures of the two behaviors. This question may be more properly explored within the oculomotor realm (34).

The U- or inverted U-shaped trajectories over age of visual tracking and SRT performances were overall reminiscent of known brain size changes (31, 40). However, the maximum whole brain weight or volume is attained by late adolescence, while the inflection points of our performance metrics were estimated to be located after young adulthood. Thus, a link between brain structures and these functional capacities cannot be drawn at such a gross level of comparison. On the other hand, structure–function correlations may be elucidated at a regional microscopic level since brain changes in development and aging are not uniform across regions or by mechanism within a region (40–42). Changes in interregional connectivity also take place during development and aging (43, 44). Our approach to model performance changes over the entire age range, rather than separately modeling development and aging effects, could also have masked effects related to inhomogeneity in brain development and aging. However, a justification for our approach may be found in the possibility that regions that are late to mature are more vulnerable to age-related declines (21, 42).

Our small sample size precluded us from deriving insight into inter-individual differences. For example, inter-individual variability was evident at any age in both visual tracking and SRT metrics, but it was not possible to examine whether the extent of inter-individual variability is comparable across ages or is reduced for a particular age range. Additionally by the cross-sectional nature of this study, it was not possible to examine whether individual standings among similarly aged peers were fluid or generally fixed through development or aging. Also limited by the sample size as well as the cross-sectional design, we were not able to examine potential differential timing of maturity between visual tracking and SRT performances. Finally, as we focused on maintenance of predictive visual tracking by utilizing a specialized visual stimulus, responses to transients or other varieties of stimuli were not studied.

Visual tracking performance metrics may provide visuomotor signatures of abnormal neurological or cognitive states, including schizophrenia, Parkinson’s disease, traumatic brain injury, and sleep deprivation (7, 28, 29, 45). These metrics may have unique utility given that visual tracking performance could present indications of abnormality independent from those which can be inferred from reaction time performance. Age is an important covariate for visual tracking performance when examined across the lifespan, especially for a pediatric population. Further understanding of age-dependent variations in normal visual tracking behavior is necessary.

## Ethics Statement

The protocol was reviewed and approved by the Weill Cornell Medical College Institutional Review Board. Prior to data collection, written informed consent by adult subjects, or legal guardians of minor subjects with the minors’ assent, was obtained in accordance with the Declaration of Helsinki.

## Author Contributions

JM and JG designed experiments and oversaw data collection and analysis. JM, LS, and UR contributed to data management and conducted statistical analyses. JM drafted the manuscript. All authors contributed to the interpretation of data and to revising the work.

## Conflict of Interest Statement

JG is director of Sync-Think, Inc., and the inventor of U.S. patent 7,384,399. JM holds stock option in Sync-Think. LS and UR have a consulting arrangement with Sync-Think. JM, UR, and JG are inventors of pending patent application PCT/US2016/027923 related to the subject matter described in this article. The authors declare no other potential conflicts of interest with respect to the research, authorship, and/or publication of this article.

## Abbreviations

SDRE, SD of radial errors; SDTE, SD of tangential errors; SRT, simple reaction time.
